# Smooth Muscle Specific Overexpression of p22^phox^ Potentiates Carotid Artery Wall Thickening in Response to Injury

**DOI:** 10.1155/2015/305686

**Published:** 2015-04-05

**Authors:** Michael R. Manogue, Justin R. Bennett, Drury S. Holland, Chung-Sik Choi, Douglas A. Drake, Mark S. Taylor, David S. Weber

**Affiliations:** Department of Physiology, University of South Alabama, Mobile, AL 36688, USA

## Abstract

We hypothesized that transgenic mice overexpressing the p22^phox^ subunit of the NADPH oxidase selectively in smooth muscle (Tg^p22smc^) would exhibit an exacerbated response to transluminal carotid injury compared to wild-type mice. To examine the role of reactive oxygen species (ROS) as a mediator of vascular injury, the injury response was quantified by measuring wall thickness (WT) and cross-sectional wall area (CSWA) of the injured and noninjured arteries in both Tg^p22smc^ and wild-type animals at days 3, 7, and 14 after injury. Akt, p38 MAPK, and Src activation were evaluated at the same time points using Western blotting. WT and CSWA following injury were significantly greater in Tg^p22smc^ mice at both 7 and 14 days after injury while noninjured contralateral carotids were similar between groups. Apocynin treatment attenuated the injury response in both groups and rendered the response similar between Tg^p22smc^ mice and wild-type mice. Following injury, carotid arteries from Tg^p22smc^ mice demonstrated elevated activation of Akt at day 3, while p38 MAPK and Src activation was elevated at day 7 compared to wild-type mice. Both increased activation and temporal regulation of these signaling pathways may contribute to enhanced vascular growth in response to injury in this transgenic model of elevated vascular ROS.

## 1. Introduction

Current strategies to treat occlusive vessel disease are pharmacological interventions, surgical bypass, and percutaneous transluminal coronary angioplasty (PTCA) with stent implantation. However, PTCA remains limited by restenosis, which occurs to a clinically significant degree in as many as 30% of all balloon angioplasty cases and an estimated 20% of all stent implantations [1]. With the advent of coated stents (sirolimus, paclitaxel), restenosis has been minimized; however, their use is limited by in-stent thrombosis, an additional complication occurring in a significant percentage of patients, typically with catastrophic results [2].

A growing body of evidence has linked reactive oxygen species (ROS) to vascular pathology. Recent data have demonstrated an important role for ROS in the vascular repair process that occurs in response to therapeutic interventions such as PTCA [3, 4] (see [5] for review). ROS initially received attention as a potential therapeutic target when an attenuated neointimal formation was observed in patients exposed to various antioxidant treatments [6]. While they act as a cytotoxic weapon of the immune system at elevated concentrations, ROS at lower concentrations act as secondary messengers affecting downstream protein activation in vascular smooth muscle cell (VSMC) migration and growth in particular [7, 8].

The quantity of ROS produced within the vascular wall is proportional to the degree of injury induced by experimental angioplasty [9]. While there are numerous potential sources of ROS in the vasculature, including neutrophil NADPH oxidase, xanthine oxidase, and the mitochondria, the vascular NADPH oxidases have emerged as the most important source of ROS in the context of vascular wall lesion development and response, accounting for 95% of ROS in the vascular wall [10, 11]. The membrane bound portion of the vascular NADPH oxidase is comprised of a functional subunit Nox1, Nox2, or Nox4, responsible for electron transport and superoxide anion radical production, and the p22^phox^ subunit. This is common to all Nox variants except Nox5, identified exclusively in human VSMCs, which does not require p22^phox^ [12]. Genetic models of p22^phox^ overexpression in VSMCs display increased levels of Nox1 particularly in those cells, suggesting a stabilizing role for p22^phox^ [13]. The p22^phox^ overexpressing transgenic mice used in our current study have been shown to upregulate p22^phox^ exclusively in smooth muscle [13], and this resulted in increased angiotensin II-induced H_2_O_2_ [8].

Studies using balloon injury in wild-type rat carotid arteries have shown that expressions of p22^phox^ and Nox1 are upregulated following balloon injury [14], and a previously reported arterial injury model demonstrated attenuated neointima formation in response to mechanically induced injury in Nox2 deficient mice [15]. A recent study has investigated the effect of VSMC-specific Nox1 overexpression in an injury model but did not characterize in vivo signaling [16]. In human studies, coronary arteries from explanted hearts have elevated ROS levels in atherosclerotic plaques that localize with increased expression of both p22^phox^ and Nox2 [17]. Additionally, intravascular imaging correlated increased ROS with p22^phox^ expression at site of expansive coronary remodeling [18]. Thus, while an association between ROS and vascular injury has been established, minimal data illustrating the in vivo relevance of specifically targeted VSMC-specific ROS overexpression in the context of experimental vascular injury is available.

Many signaling pathways have been implicated in the VSMC response to elevated ROS levels; however, their activation has not been evaluated following vascular injury in vivo. The present study focuses on Akt/protein kinase B (PKB), p38 mitogen activated protein kinase (MAPK), and Src involvement in the arterial injury response in vivo. In vitro evidence has demonstrated that Akt, p38 MAPK, and Src require H_2_O_2_ for their activation [19–21]. Furthermore, each has been shown to regulate VSMC proliferation and migration, processes necessary for vessel wall thickening [22]. To date, the temporal regulation of ROS-sensitive signaling pathways in vivo has not been well characterized in animal models of vascular injury.

In this study, we used unique transgenic mice that overexpress the NADPH oxidase subunit p22^phox^ selectively in smooth muscle, allowing us to determine the specific contribution of elevated VSMC ROS on the vascular response to injury. We tested the hypothesis that overexpression of p22^phox^ resulting in increased ROS in the vascular wall potentiates the vascular injury response due to increased ROS-dependent protein activation in vivo.

## 2. Materials and Methods

### 2.1. Animals

Mice used in the current study overexpress p22^phox^ specifically in their smooth muscle [13]. To generate these animals, p22^phox^ was cloned downstream of the *α*-actin SMP-8 promoter and upstream of a SV40 poly-A fragment, as previously described [8]. Founder mice were then backcrossed 10 generations to the C57BL/6J strain. For the current experiments, mice that were heterozygous for p22^phox^ overexpression in smooth muscle (Tg^p22smc^) and their negative littermate controls were used. Studies were completed in adult male mice, aged 12–16 weeks. All animal procedures were conducted with approval of the IACUC of the University of South Alabama and in accordance with the* Guide for the Care and Use of Laboratory Animals* (NIH Publication Number 85-23, revised 1996).

### 2.2. Surgical Procedure

Animals were anesthetized with a mixture of ketamine (75 mg/kg) and xylazine (5 mg/kg) via intraperitoneal injection. The left external carotid artery was isolated by blunt dissection and carefully dissected free from the nerve with forceps via a midline incision. 7-0 silk braided sutures were looped proximally around the common, internal, and external carotid branches to temporarily occlude flow. A nylon wire catheter with a diameter 0.30 mm was inserted 5 mm proximally into the common carotid artery via an arteriotomy in the external carotid to ensure denudation of the endothelium. Upon removal of the catheter, the external carotid artery was permanently ligated proximal and distal to the arteriotomy using 7-0 silk sutures and flow was reestablished in the common and internal carotid. In some studies apocynin (0.25 mg/mL in drinking water) was administered for 2 days prior to the procedure and continued until tissue harvest [23].

### 2.3. Tissue Harvest and Morphometric Analysis

Animals were administered a pentobarbital overdose (100 mg/kg i.p.) and the thoracic cavity opened to expose the heart. A 25-gauge winged tip catheter infusion kit (Terumo) was introduced into the left ventricle, exsanguination was completed via a saline flush, and the vasculature was fixed with 10% formaldehyde solution at physiologic pressure. After tissue fixation, an en bloc excision of the bilateral carotid arteries was performed and samples were embedded in paraffin. Multiple tissue cross sections (5 *μ*m) were stained with hematoxylin and eosin and digital images of the injured vessel and the contralateral uninjured vessel were obtained using a 10x objective. Sections were obtained beginning at the carotid artery bifurcation and extending 5 mm distally into the common carotid artery to ensure multiple sections were assessed within the injured artery. NIH image software was used to determine artery wall thickness and cross-sectional wall area (CSWA). Eight random measurements of the distance from the luminal border to the external lamina were averaged per animal and taken as wall thickness. CSWA was calculated by obtaining the area within the outermost elastic lamina of the vessel and subtracting the area of the vessel lumen. For each artery, multiple cross sections were assessed within the 5 mm region of injury and degree of injury was consistent throughout sequential sections.

### 2.4. Visualization of Endothelium

Isolated carotid artery segments (5 mm) were carefully opened longitudinally and pinned, luminal-side-up, on small silicone (Sylgard) blocks. Arteries were treated with a membrane permeant fluo-4 AM Ca^2+^ indicator loading solution [24]. After washing with physiological saline, blocks were placed upside down into a glass-bottom chamber where they were separated from the bottom by 100 *μ*m diameter pins. The chamber was placed on the stage of an inverted spinning-disk laser confocal microscope (PerkinElmer RS-3) and Ca^2+^ dependent fluorescence of endothelial cells along the intima was imaged (ex. 488 nm, em. 510 nm, 20x objective, at 25°C). Images were captured with Ultraview software. The presence of intact endothelial cells is confirmed by dye uptake.

### 2.5. Superoxide Anion Radical Detection Using Dihydroethidium (DHE)

Carotid arteries were harvested, embedded in OCT, and snap frozen. 7 *μ*m sections were cut using a cryostat and stained with DHE (10 uM) in PSS. Tissue sections were visualized (ex. 510 nm, em. 595 nm, 40x objective) using Nikon80i upright fluorescent microscope and captured using Nikon Elements software.

### 2.6. Immunoblotting

Carotid arteries were harvested and snap frozen in liquid nitrogen. Tissues were then minced and lysed and proteins harvested as previously described [21]. Antibodies recognizing phosphorylated Akt (ser473), p38 MAPK (tyr180/thr182), and Src (tyr416) were obtained from Cell Signaling Technology. Immunoblot densitometry was quantified using UN-SCAN-IT software (Silk Scientific). Protein expression was determined in the injured (left) carotid artery (LC) and the uninjured (right) carotid artery (RC) for each mouse. Cumulative data are presented as the ratio of expression in the injured (left) carotid artery (LC) over the uninjured (right) carotid artery (RC) to normalize potential variability between mice.

### 2.7. Statistical Analysis

All data were presented as mean ± SEM. Depending upon design, unpaired *t*-tests or analysis of variance was completed. A Newman-Keuls multiple-comparison posttest was performed following analysis of variance. In all cases, a *P* value <0.05 was selected to denote statistical significance between groups.

## 3. Results

### 3.1. The Increase in Vascular Wall Thickness and Cross-Sectional Wall Area following Injury Is Exacerbated in Mice Overexpressing p22^phox^

**Figure 1:**
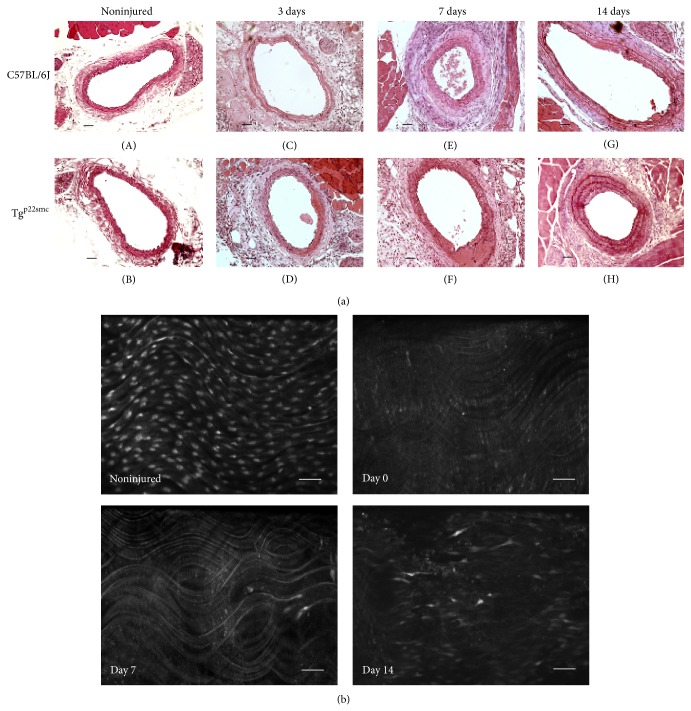
(a) Hematoxylin and eosin stained cross sections of carotid arteries harvested at selected time points following injury in C57BL/6J and Tg^p22smc^ mice. Representative sections from noninjured arteries ((A) and (B)) and carotid arteries 3 days after injury ((C) and (D)), 7 days after injury ((E) and (F)), and 14 days after injury ((G) and (H)) are shown. (b) Representative luminal images of fluo-4 treated sections of isolated carotid artery segments from a noninjured artery and injured arteries at days 0, 7, and 14 days following vascular injury. All images were obtained at a magnification of 200x and the scale bar is equal to 20 *μ*m.

**Figure 2:**
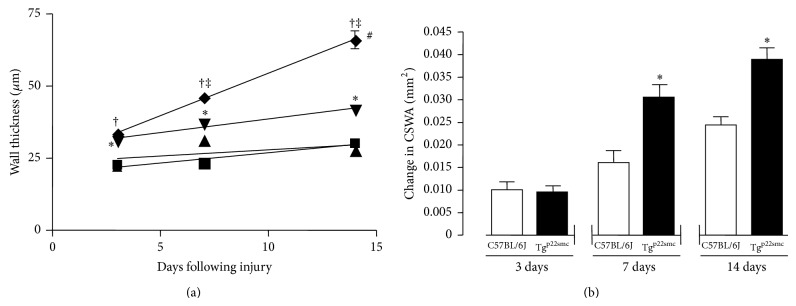
(a) Mean wall thickness in the injured and noninjured, contralateral arteries following wire injury plotted against time in the Tg^p22smc^ (◆: injured; ▲: control) and C57BL/6J (▼: injured; ■: control) mice. ^*^Significant increase in mean wall thickness in C57BL/6J injured arteries versus C57BL/6J noninjured arteries at the same time points (*P* < 0.001). ^†^Significant increase in mean wall thickness in Tg^p22smc^ injured arteries versus Tg^p22smc^ noninjured arteries at the same time points (*P* < 0.001). ^‡^Significant increase in mean wall thickness in Tg^p22smc^ injured arteries versus C57BL/6J injured arteries at the same time points (*P* < 0.001). Linear regression analysis indicates the rate of the change in wall thickness in injured carotid arteries in Tg^p22smc^ versus C57BL/6J mice (^#^ *P* < 0.04). (b) Mean change in carotid artery cross-sectional wall area (CSWA) 3, 7, and 14 days after injury in Tg^p22smc^ mice (*filled bars*) compared to C57BL/6J (*open bars*). ^*^Significant increase in mean CSWA in Tg^p22smc^ injured arteries versus C57BL/6J injured arteries at the same time points (*P* < 0.001). Values represent mean ± SEM for a minimum of 8 animals per group.

To determine the vascular response to injury in the presence of increased VSMC ROS, we obtained histological sections of each artery in axial cross section. Hematoxylin and eosin images were used to observe and quantify changes in arterial morphology following wire injury (Figure 1(a)). En face confocal microscopy images were used to confirm successful endothelial removal and establish the subsequent time course of repair following wire-injury. Substantial reendothelialization occurred 14 days following injury (Figure 1(b)) and the endothelium appears fully regenerated 21 days following injury (data not shown).  Figure 2(a) reports the cumulative data of wall thickness measurements following carotid injury in both groups of mice. Uninjured arteries in p22^phox^ and wild-type mice showed no significant difference in wall thickness throughout the time course of injury. At 3 days after injury, both Tg^p22smc^ and wild-type groups showed small but significant increases in the mean wall thickness when comparing injured to uninjured arteries, but there was no significant difference in the mean arterial wall thickness between the two injured groups at day 3. At 7 days following injury, both Tg^p22smc^ and wild-type groups showed increased wall thickness when comparing uninjured arteries to injured arteries, but, in contrast to day 3, the difference in wall thickness was significantly greater in Tg^p22smc^ injured vessels than wild-type injured vessels (46.1 ± 2.6 versus 37.1 ± 2.8 *μ*m). At day 14 after injury, both groups of mice demonstrated increased wall thickness, and, similar to day 7, wall thickness was significantly greater in Tg^p22smc^ mice compared to wild-type mice (66.0 ± 10 versus 41.8 ± 2 *μ*m) (Figure 2(a)). Moreover, in addition to demonstrating a significantly larger maximal wall thickness measured at 14 days following injury, the rate of change in wall thickness during the progression of injury was significantly accelerated in the Tg^p22smc^ group (slopes 2.9 ± 0.08, *r* ^2^ = 1.0 versus 0.95 ± 0.2, *r* ^2^ = 0.95).

The average change in cross-sectional wall area (CSWA) was calculated in each animal by comparing the uninjured artery to the injured artery.  Figure 2(b) shows the mean increases in CSWA for both wild-type and Tg^p22smc^ mice at the designated time points. There were modest increases in CSWA in both groups at the 3-day time point, but they were not significantly different between the groups. We observed that the increase in medial mass as measured by CSWA was exacerbated at days 7 and 14 in Tg^p22smc^ mice compared to wild-type mice.

### 3.2. ROS Production Is Elevated in Carotid Arteries following Injury

**Figure 3:**
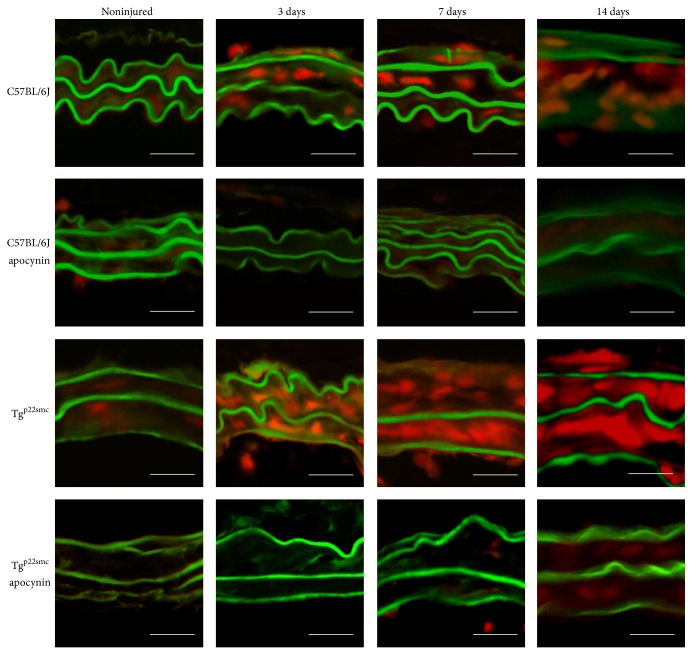
Representative dihydroethidium (red) stained cross sections of carotid arteries harvested at 3, 7, and 14 days following injury in nontreated or apocynin treated C57BL/6J and Tg^p22smc^ mice. Scale bar is equal to 20 *μ*m.

**Figure 4:**
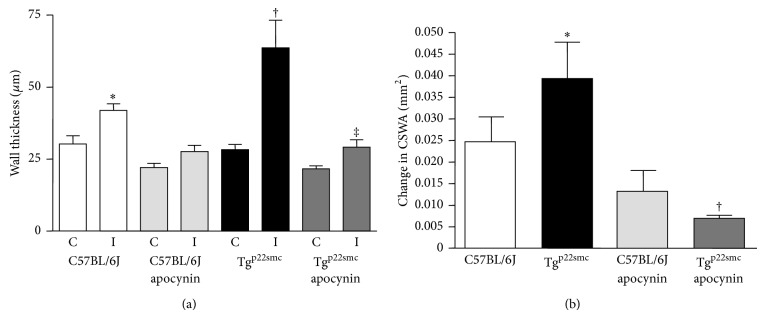
(a) Effect of apocynin treatment on wall thickness 14 days after injury in Tg^p22smc^ and C57BL/6J mice. ^*^Significant increase in mean wall thickness in C57BL/6J injured arteries versus C57BL/6J noninjured arteries (*P* < 0.001). ^†^Significant increase in mean wall thickness in Tg^p22smc^ injured arteries versus Tg^p22smc^ noninjured arteries (*P* < 0.001). ^‡^Significant decrease in mean wall thickness versus Tg^p22smc^ injured arteries from nontreated mice (*P* < 0.001). (b) Effect of apocynin treatment on the change in carotid artery cross-sectional wall area (CSWA) 14 days after injury in Tg^p22smc^ and C57BL/6J mice. ^*^Significant increase in mean CSWA in Tg^p22smc^ injured arteries versus C57BL/6J injured arteries (*P* < 0.05). ^†^Significant decrease in CSWA versus Tg^p22smc^ injured arteries from nontreated mice (*P* < 0.05). Values represent mean ± SEM for a minimum of 6 animals per group.

DHE staining was completed to examine temporal changes in ROS production in the arterial wall following wire injury. While ROS increases following injury in both WT and Tg^p22smc^ mice, the levels were higher throughout the time course following injury in the Tg^p22smc^ mice and correlated with greater increases in wall thickness. Continuous apocynin treatment prevented increased ROS production following injury in both groups (Figure 3). Apocynin also limited the wall thickening (Figure 4(a)) and increases in CSWA (Figure 4(b)) measured 14 days following injury in both groups, suggesting that vascular ROS was a key contributor to the remodeling response.

### 3.3. VSMC p22^phox^ Overexpression Enhances the Activation of Akt, p38 MAPK, and Src following Vascular Injury

As our data indicated that VSMC-specific p22^phox^ overexpression exacerbated the vascular response to injury, we wanted to identify potential signaling mechanisms associated with this observation and determine the time course of their activation following injury. Carotid arteries were harvested at select time points following injury and protein activation was assessed by phosphorylation. We tested Akt and p38 MAPK, as well as their upstream regulator Src, as the activation of each has been shown to be ROS-dependent in several cell types including VSMCs [22]. Importantly, neither total protein expression nor phosphorylation of any of these proteins differed between noninjured wild-type and Tg^p22smc^ arteries (data not shown).

**Figure 5:**
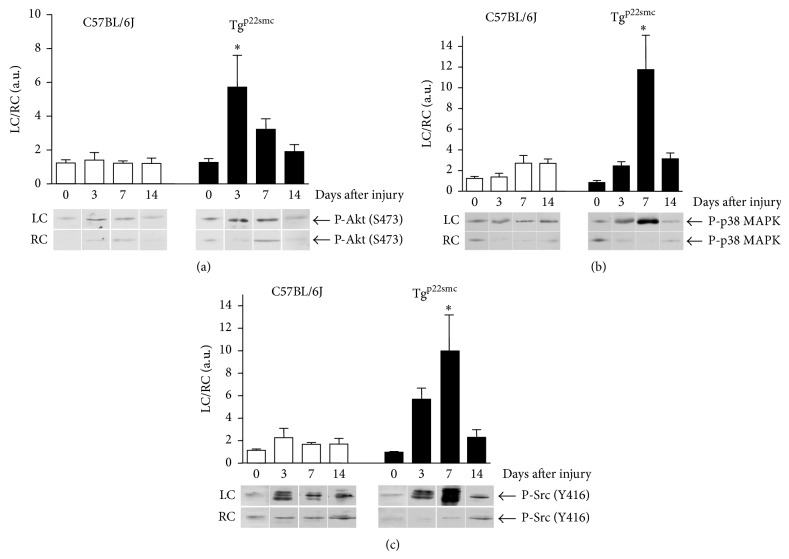
Akt, p38MAPK, and Src phosphorylation following carotid injury in Tg^p22smc^ and C57BL/6J mice. The phosphorylation of Akt (ser473) (a), p38MAPK (tyr180/thr182) (b), and Src (tyr416) (c) was determined in both injured (LC) and noninjured (RC) carotid arteries collected at days 0, 3, 7, and 14 following injury. Representative immunoblots from the LC and RC are shown. Cumulative data of the LC/RC ratio are represented in the bar graphs. Values represent mean ± SEM for a minimum of 6 animals per group. ∗ A significant increase in phosphorylation following injury in carotid arteries from Tg^p22smc^ mice compared to C57BL/6J mice at the same time point (*P* < 0.05). Total expression of Akt, p38MAPK, and Src did not change significantly after injury and basal phosphorylation of Akt, p38MAPK, and Src was not different between noninjured arteries in wild-type and Tg^p22smc^ mice.

Akt phosphorylation in wild-type mice was not appreciably increased following injury at any of the timepoints tested (Figure 5(a)). In contrast, in Tg^p22smc^ mice, the level of Akt phosphorylation at day 3 was increased (5.7 ± 1.9 fold), remained elevated at 7 days after injury (3.2 ± 0.6 fold), and returned to near baseline by 14 days after injury (1.9 ± 0.4). In wild-type mice, p38 MAPK is moderately activated at days 7 and 14 following injury (2.7 ± 0.7 and 2.7 ± 0.4 fold) (Figure 5(b)). The degree of p38 MAPK activation was exacerbated following wire injury in Tg^p22smc^ mice when contrasted to activation in wild-type mice, increasing moderately at day 3 (2.5 ± 0.4 fold), robustly elevated at day 7 (11.7 ± 3.3 fold), and remaining slightly increased at day 14 (3.2 ± 0.6 fold). The activation of Src in wild-type mice is slightly increased at 3 days after injury (2.3 ± 0.8 fold), and this modest activation is maintained at both 7 and 14 days after injury (1.7 ± 0.1- and 1.7 ± 0.5-fold) (Figure 5(c)). In Tg^p22smc^ mice wire injury resulted in strong activation of Src by 3 days following injury (5.7 ± 1.0-fold), a further increase at 7 days after injury (10.0 ± 3.2-fold), and a return toward baseline by day 14 (2.3 ± 0.7-fold). Taken together, our in vivo signaling studies suggest that enhanced ROS-sensitive protein activation in Tg^p22smc^ mice may be the mechanism underlying the exacerbated vascular wall thickening in these mice following wire injury.

## 4. Discussion

In these studies we utilized transgenic mice to examine the effect of VSMC-specific overexpression of p22^phox^ in mediating the vascular response to carotid injury. Our findings indicate that the elevated basal levels of ROS in the transgenic animals significantly exacerbated both the rate and the maximal extent of wall thickening following injury. Our results also, for the first time, establish a correlation between the temporal activation of Akt, p38 MAPK, and Src with wall thickening and suggest their significant involvement in the regulation of the VSMC response to injury in vivo.

Previous studies utilizing these mice and other transgenic mice engineered to have VSMC-specific alterations in NADPH oxidase subunit expression have suggested that while elevated vascular ROS levels are present, the mice demonstrate normal vascular structure and function in the absence of any intervention [8, 25]. In Tg^p22smc^ mice, increased superoxide anion radical is released mainly intracellularly, these animals have basal compensatory increases in eNOS and ecSOD expression, and basal NO production is elevated [13]. Thus the lack of basal vascular alterations in carotid structure in noninjured vessels in the current study is consistent with previous studies. In our studies, maximal changes in both wall thickness and CSWA measured 14 days following wire injury of the carotid artery were significantly potentiated in the p22^phox^ mice compared to wild-type animals, implicating elevated vascular ROS as a key mediator. This was confirmed by the ability of apocynin to prevent vascular remodeling in our studies. Our findings are consistent with previous studies in which the antioxidant N-acetylcysteine (NAC) reduced neointimal thickening in response to balloon injury in rabbits [26] as well as studies in which intravascular adenoviral-mediated delivery of superoxide dismutase (SOD) and catalase decreased oxidative stress and restenosis in response to balloon injury in rabbits [27, 28]. In clinical trials, AG-1067, a synthetic probucol and potent antioxidant, reduced restenosis in response to PTCA [6]. Moreover, a decrease in p47^phox^ and p67^phox^, subunits of the NADPH oxidase that are required for its activation, was associated with decreased neointima formation after carotid ligation in C57BL/6 mice [29]. Lee et al. recently reported decreased neointimal formation following wire injury of the femoral artery in Nox1 knockout mice, but, surprisingly, transgenic mice with VSMC-specific overexpression of the NADPH subunit Nox1 did not demonstrate significantly more neointimal formation [16]. This group postulates that additional Nox1 activity in these mice was not distinguishable from the increased Nox1 activity that occurs in wild-type mice following injury. An alternative explanation may be that in the Nox1 overexpressing mice there is no increase in p22^phox^ expression [25] which is required for the stabilization of Nox1 and, thus, NADPH oxidase activation [30]. Also, in our Tg^p22smc^ mice there may be activation of multiple Nox isoforms contributing to the wall thickening following injury.

By quantifying injury at several timepoints following intervention in our studies, we were able to demonstrate that p22^phox^ overexpression potentiated the temporal progression of vascular wall thickening following injury and correlated to the activation of Akt, p38 MAPK, and Src, proteins relevant to VSMC proliferation, migration and survival [22, 31], processes necessary for wall remodeling after injury, including neointima formation and medial thickening. Thus, our results demonstrating their activation are not surprising. Our studies also demonstrate a timeline for endothelial regrowth following wire injury of approximately 14 days, which correlated with the return of Akt, p38 MAPK, and Src activation close to basal levels.

A role for Akt in mediating vascular remodeling following injury has been established previously. Lipoxygenase inhibition blocked cell cycle progression and decreased VSMC proliferation and balloon angioplasty-induced intimal hyperplasia in rabbits, and this effect was associated with reduction in Akt and ERK1/2 MAPK activation [32]. Adenovirus-mediated delivery of constitutively active Akt reversed the antiproliferatory, antimigratory, and antirestenotic effects of the protooncogene C-Cbl in a rat model of balloon injury in vivo [33]. Of clinical relevance, Akt is directly proximal to mammalian target of rapamycin (mTOR) in the proliferative signaling cascade. Rapamycin (sirolimus) is one of the two primary active pharmacological agents contained in drug-eluting stents used during PTCA. In the long term RAVEL clinical trial, 5-year rates in absentia of revascularization was 99.2% for sirolimus eluting stents while the rate in patients receiving bare metal stents was 74% [34].

There have been several previous studies which link the activation of p38 MAPK with vascular remodeling. PDGF-BB has been shown to initiate migration in VSMC in a dose-dependent manner by activating the p38 MAPK/HSP27 pathway [35, 36]. p38 MAPK has also been implicated in restenosis [37–39]. Although the exact mechanism by which p38 activation promotes restenosis is incompletely understood, one possibility is that it does so by regulating the expression and/or activation of ECM-degrading enzymes such as matrix metalloproteases (MMPs). MMP-2 and MMP-9 have been shown to play a role in neointimal formation [40]. Chen et al. have shown that under hypoxic conditions both p38 and MMP-2 activation occurred prior to VSMC migration. When p38 activation was inhibited with SB203580 both MMP-2 activation and VSMC migration failed to occur [41]. Recent findings indicate that increased MMP-9 activity, leading to N-cadherin shedding, has been shown to be dependent upon Nox1 transactivation of epidermal growth factor receptor (EGFR) during thrombin induced VSMC migration in vitro. Interestingly, this mechanism requires the activation of Src by the Nox1 to stimulate EGFR [42].

Our previous studies have established the nonreceptor linked tyrosine kinase Src as the most proximal ROS-dependent kinase in mediating PDGF-induced VSMC migration via a Src/PDK1/PAK pathway [21] and Nox subunit inhibition by VAS2870 has linked Src mediated PDGF-induced VSMC chemotaxis to NADPH-dependent ROS generation [43]. A recent report indicates that one mechanism by which Src may contribute to neointima formation is via the control of microRNA expression, in particular of miR-143/145, which regulates the phenotypic switch of VSMC from a contractile to a synthetic and promigratory phenotype [44]. Giannoni et al. [45] have recently proposed a model of ROS-dependent Src signaling that hypothesizes that Src is a key mediator of invadopodia and lamellipodia formation associated with cell chemotaxis. While our findings are consistent with these previously observed roles for Src, they are the first to implicate Src in vivo as potentially a key mediator in ROS-mediated response to vascular injury. Since Src is a known upstream regulator of both Akt and p38MAPK, it may integrate the upstream activation of both proteins in this injury model and thus would be a logical target for study as a potential target for the prevention of restenosis following PTCA.

Taken together, the results from our in vivo protein studies demonstrating significantly greater activation of these kinases in response to vascular injury in the Tg^p22smc^, a high ROS model, are in agreement with previous observations which have suggested the regulation of these proteins is altered by ROS [23, 46]. Our study provides in vivo evidence of the link between the ROS-dependent activation of these kinases during vascular wall thickening in response to injury as well as the first direct characterization of the temporal regulation of their activation in vivo in a mouse model of elevated VSMC ROS levels due to increased p22^phox^ expression.

One limitation of our current findings is that, due to complexities of the vascular repair process, future studies will be required to elucidate signaling mechanisms downstream from Akt, p38MAPK, and Src for therapeutic design. A potential mechanism could be the ROS-dependent regulation of Nf-*κ*B activity. At 3 days following injury, we observed decreased IkB-*α* levels, indicative of increased Nf-*κ*B activity. Since both the degree and temporal pattern of activation were similar between wild-type and Tg^p22smc^ mice during our time course, we cannot conclude that Nf-*κ*B is the distinguishing downstream signaling target that accounts for the exacerbated remodeling in the Tg^p22smc^ mice (data not shown). Additional studies of ROS-dependent mechanisms may also require earlier and later time points following injury to fully appreciate the contributions of ROS in the processes of apoptosis and constrictive remodeling, both of which contribute significantly to outcomes following vascular injury repair.

## 5. Conclusions

Our results demonstrate that locally elevated vascular ROS levels in vivo are associated with greater vascular wall thickening and potentiated Akt, p38 MAPK, and Src activation. A more detailed knowledge of ROS-dependent molecular pathways in vivo including their temporal regulation is becoming more relevant as systemic, relatively nonspecific antioxidant therapeutic strategies have yielded no apparent benefit in reducing vascular disease or remodeling in large scale clinical trials [47–49]. Moreover, both sirolimus and paclitaxel, compounds effective in lowering restenosis when used in coated stents during PTCA, have also been reported to increase ROS levels leading to impaired endothelial function and changes in NO synthesis [50, 51]. Thus, future successful cardiovascular therapies based on oxidative stress will likely target localized specific signaling pathways activated by ROS, rather than targeting ROS production nonselectively. Our results indicate that VSMC NADPH oxidase-derived ROS potentiate the vascular response to experimental angioplasty along with increased activation of Akt, p38 MAPK, and Src, which renders these pathways potential targets for future therapies aimed at reducing restenosis.
